# Accelerating newborn survival in Ghana through a low-dose, high-frequency health worker training approach: a cluster randomized trial

**DOI:** 10.1186/s12884-018-1705-5

**Published:** 2018-03-22

**Authors:** Patricia P. Gomez, Allyson R. Nelson, Amos Asiedu, Etta Addo, Dora Agbodza, Chantelle Allen, Martha Appiagyei, Cynthia Bannerman, Patience Darko, Julia Duodu, Fred Effah, Hannah Tappis

**Affiliations:** 1 Jhpiego/Baltimore, 1615 Thames Street, Baltimore, MD 21232 USA; 2 Jhpiego/Liberia, UN Drive, OPP Rock Compound, Mamba Point, Monrovia, Liberia; 3 Jhpiego Ghana, 14 Ollenu Street, East Legon, Accra, Ghana; 4 Ghana Health Service, Private Mail Bag, Ministries, Accra, Ghana

**Keywords:** Pregnancy, Labor, Birth, Stillbirth, Newborn, Resuscitation, Training, Skills, Skilled birth attendant, Mentor

## Abstract

**Background:**

Newborn deaths comprise nearly half of under-5 deaths in Ghana, despite the fact that skilled birth attendants (SBAs) are present at 68% of births, which implies that evidence-based care during labor, birth and the immediate postnatal period may be deficient. We assessed the effect of a low-dose, high-frequency (LDHF) training approach on long-term evidence-based skill retention among SBAs and impact on adverse birth outcomes.

**Methods:**

From 2014 to 2017, we conducted a cluster-randomized trial in 40 hospitals in Ghana. Eligible hospitals were stratified by region and randomly assigned to one of four implementation waves. We assessed the relative risks (RRs) of institutional intrapartum stillbirths and 24-h newborn mortality in months 1–6 and 7–12 of implementation as compared to the historical control period, and in post-intervention facilities compared to pre-intervention facilities during the same period. All SBAs providing labor and delivery care were invited to enroll; their knowledge and skills were assessed pre- and post-training, and 1 year later.

**Results:**

Adjusting for region and health facility type, the RR of 24-h newborn mortality in the 40 enrolled hospitals was 0·41 (95% CI 0·32–0·51; *p* < 0.001) in months 1–6 and 0·30 (95% CI 0·21–0·43; *p* < 0·001) in months 7–12 compared to baseline. The adjusted RR of intrapartum stillbirth was 0·64 (95% CI 0·53–0·77; *p* < 0·001) in months 1–6 and 0·48 (95% CI 0·36–0·63; *p* < 0·001) in months 7–12 compared to baseline. Four hundred three SBAs consented and enrolled. After 1 year, 200 SBAs assessed had 28% (95% CI 25–32; *p* < 0·001) and 31% (95% CI 27–36; *p* < 0·001) higher scores than baseline on low-dose 1 and 2 content skills, respectively.

**Conclusions:**

This training approach results in a sustained decrease in facility-based newborn mortality and intrapartum stillbirths, and retained knowledge and skills among SBAs after a year. We recommend use of this approach for future maternal and newborn health in-service training and programs.

**Trial registration:**

Retrospectively registered on 25 September 2017 at Clinical Trials, identifier NCT03290924.

**Electronic supplementary material:**

The online version of this article (10.1186/s12884-018-1705-5) contains supplementary material, which is available to authorized users.

## Background

The newborn mortality rate decreased by 47% worldwide between 1990 and 2015, from 36 to 19 deaths per 1000 live births, but the rate has declined more slowly in most low-resource settings, and is still far from the Sustainable Development Goal of less than 12 newborn deaths per 1000 live births by 2030.^1^ Nearly half of newborn deaths take place during the first day of life [1]. Intrapartum-related events are now the third leading cause of all deaths among children under age 5 (U5 mortality) [2]. Rates of intrapartum stillbirths are a measure of the quality of care during labor, and newborn mortality in the 24 h following birth can reflect the quality of care during labor as well as immediate postnatal care [3].

Ghana’s U5 mortality rate is 60/1000 live births and the newborn mortality rate is 29/1000 live births. Thus, newborn mortality represents 48% of U5 mortality despite the fact that 68% of births occur with a skilled birth attendant (SBA)^2^ present [4]. The country’s stillbirth rate is estimated at 22/1000 total births, although this estimate is not disaggregated by fetal deaths prior to labor or intrapartum stillbirths, and accurate information about the incidence of intrapartum stillbirth is lacking [5]. Two major causes of newborn deaths in Ghana, asphyxia (23%) and sepsis (32%), [6] could be prevented by increasing the use of evidence-based interventions during labor, birth, and the immediate postnatal period. The Ghana National Newborn Health Strategy and Action Plan 2014–2018 describes the country’s goal to reduce institutional newborn mortality by at least 35% by 2018 through training of least 90% of SBAs in essential newborn care and basic resuscitation [6].

In-service training for SBAs is one of the most common interventions to address lack of knowledge and skills. However, these training interventions are seldom evaluated for effectiveness in improving learning or performance [7]. A literature review of effective in-service training described techniques that may improve learning outcomes and result in changes in performance [8]. The review found that didactic instruction often results in improved knowledge but no improvement in clinical practice. In contrast, the use of interactive techniques, including clinical simulation, case-based learning, hands-on practice with anatomic models, and immediate feedback on performance, results in greater improvement in knowledge and/or clinical practice than do passive techniques such as lecture or reading. Targeted, repeated training opportunities are preferable to one-time training and lead to the use of new skills in the clinical setting. Workplace learning may be superior for skill acquisition, and using multiple types of media can deliver training more efficiently. Thus, learning at the job site coupled with ongoing, repeated exposure to content is associated with greater improvement in clinical performance and health outcomes.

In collaboration with the Ghana Health Service (GHS), Jhpiego introduced interventions to reduce newborn mortality in 40 public and mission health facilities in which an ongoing quality improvement project^3^ was targeting reduction of U5 mortality. Using evidence from the literature review on in-service training, we designed and tested a low-dose, high-frequency (LDHF) training approach to update hospital-based SBAs in key evidence-based intrapartum and immediate newborn care practices, using current global guidelines [9] (Table 1).

**Table 1:** Components of low-dose, high-frequency approach. Lists the eight components that comprised the low-dose, high-frequency approach

• Two 4-day low-dose sessions (for facility skilled birth attendants)	
• 1-day peer practice coordinator training after first low-dose session	
• High-frequency practice sessions using MamaNatalie® and NeoNatalie™ anatomic models	
• mMentoring with SMS reminder messages and quizzes	
• Mentoring calls between master mentors and peer practice coordinators, and between project staff and master mentors	
• Health information officer training	
• Data collection and use training	
• Supply of simulators, newborn resuscitation equipment, and delivery sets provided at first low-dose training session	

All study sites received the full LDHF intervention that included two 4-day onsite sessions (low dose) with weekly practice sessions, SMS quizzes and reminders, and mentoring via mobile phone and onsite visits between trainings (high frequency). The low-dose sessions included competency acquisition through simulation, case-based learning, and small content packages spread over short time intervals (Table 2) [7, 10]. To foster a team-centered approach, [8] we trained all SBAs providing intrapartum and immediate postpartum care in each health facility, but limited each low-dose session to no more than ten SBAs. Thus, in large facilities more than one cycle of training was conducted to accommodate all eligible SBAs. This approach was considered low dose compared to the basic emergency obstetric and newborn care (BEmONC) training that was occurring in the country at the time of this study. The BEmONC training took place away from SBAs’ usual workplace, included only a few SBAs from a given facility, and lasted up to 10 days.

**Table 2:** Low-Dose Session Content. Lists the clinical content presented in each low-dose session

*Low-Dose Session 1:*	
• Respectful maternity care	
• Infection prevention and control	
• Clinical decision-making	
• Evidence-based support of normal labor and birth, including use of the partograph and active management of the third stage of labor	
• Immediate newborn care	
• Newborn resuscitation	
• Data collection and use; reporting of data	
*Low-Dose Session 2:*	
• Antenatal corticosteroids for anticipated preterm birth	
• Management of severe pre-eclampsia and eclampsia	
• Management of postpartum hemorrhage, including repair of lacerations	
• Prevention and treatment of maternal and newborn sepsis	
• Kangaroo mother care	
• Data collection and use; reporting of data	

We hypothesized that the LDHF approach to improving clinical skills would lead to a reduction in institutional intrapartum stillbirths and newborn mortality within 24 h of birth during the year of the intervention. We also hypothesized that the approach would result in improved and retained skills and knowledge among SBAs in maternity wards in study sites after a year.

## Methods

### Study design

**Fig. 1:**
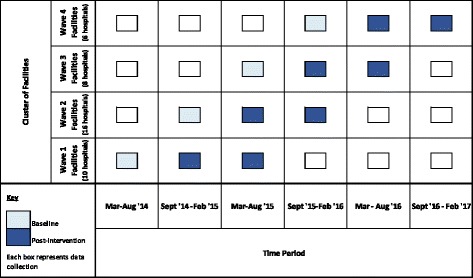
Phased implementation and data collection. Describes timing of baseline and post-intervention data collection over the four waves of facility enrollment between March 2014 and February 2017

The study was a cluster-randomized trial implemented in 40 public and mission hospitals in Ghana between March 2014 and February 2017. Initial study sites included three regional hospitals, 38 district hospitals, and one polyclinic across Upper West, Central, and Western Regions of Ghana. Each facility was enrolled for 18 months. Data on institutional intrapartum stillbirths and 24-h newborn mortality were collected retrospectively for 6 months pre-intervention (i.e., baseline) and prospectively for 12 months post-intervention at each study site (Fig. 1).

All SBAs providing labor, birth, and immediate postnatal care at the selected facilities at the time of the first low-dose session were invited to participate. Four hundred and three SBAs individually consented and were enrolled in the study. All enrolled SBAs were registered or certified midwives.

### Randomization and masking

Eligible facilities had at least three SBAs and an average of 30 or more births per month. These hospitals were stratified by region (three categories) and caseload (four categories), and then randomly assigned to one of four implementation waves. The pipeline randomization allowed for rigorous evaluation while the program was rolled out to all facilities. Waves 1 through 4 included eleven, seventeen, eight, and six facilities, respectively. Waves were unbalanced because of the stratification. Two hospitals were excluded at the time of implementation because they no longer met the inclusion criteria. Neither facilities nor participants were masked due to the nature of the intervention.

### Procedures

**Fig. 2:**
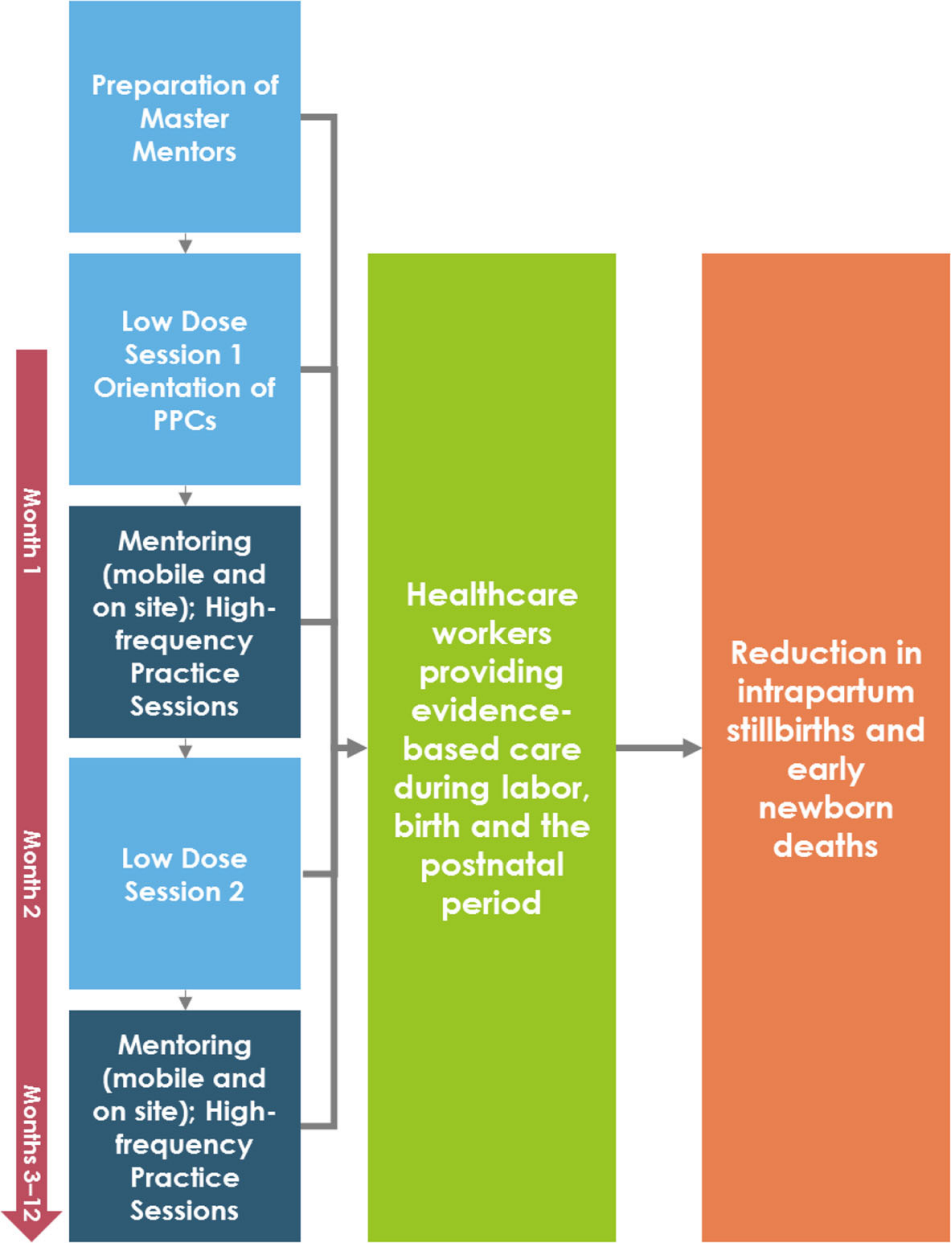
Low-dose, high-frequency training approach to reduce newborn mortality and intrapartum stillbirths in Ghana. Describes the inputs, outcomes and impact of the project

Experienced SBAs were prepared as regional master mentors (MMs) through a course in targeted BEmONC and training skills. MMs conducted the onsite, low-dose sessions and follow-up mentorship activities in collaboration with project staff. Of the 40 study facilities, 19 had a MM working within the facility, while the remaining facilities received mentoring support from MMs via mobile phone and periodic visits. During the first low-dose session at each site, two to three SBAs were trained as peer practice coordinators (PPCs) to lead the high-frequency practice sessions and were oriented to their roles in a day-long session. One month later, the second 4-day low-dose session was conducted. In the month between low-dose sessions and for 11 months afterward, SBAs carried out weekly PPC-led practice sessions with simulators and participated in mMentoring, which included mobile phone–based mentoring and onsite coaching with MMs and automated SMS reminders and quiz questions about key concepts presented during the low-dose sessions (Fig. 2).

MamaNatalie® and NeoNatalie™ simulators were given to each site to enable regular practice of targeted skills. Each site also received newborn resuscitation equipment (NeoNatalie resuscitator, penguin suction [Laerdal], and basic delivery sets). SBAs and health information officers collected service delivery data. Orientation to facility-level data collection was included in the first low-dose session for all SBAs. An additional training was provided to each facility’s health information officer and maternity unit in-charge to ensure a common understanding of indicators and data collection, quality, and use. Monthly service statistics were extracted from routine health information systems and a supplementary delivery register introduced by the project. All outcome data were verified using register reviews at each study site.

Objective structured clinical examination (OSCE) tools and knowledge tests, based on global and national guidelines, were developed to assess SBAs in management of normal birth, immediate newborn care, and maternal and newborn complications. These assessments were used before and immediately after low-dose sessions, and again 1 year later, to evaluate knowledge and skill acquisition and retention. Master mentors and project staff conducted and collected all OSCE and knowledge test data. Assessments were scanned into Captricity (Oakland, CA, USA) for data capture and cleaning.

#### Outcomes

Twenty-four hour newborn mortality was defined as the death within 24 h, or before discharge, of a newborn who breathed at birth. The institutional 24-h newborn mortality rate was defined as deaths in the first 24 h per 1000 facility live births. Intrapartum stillbirth was defined as the death of a baby who had heart tones present at the time of the mother’s admission to the facility, was born with no signs of maceration, and did not breathe at birth, and on whom resuscitation attempts were unsuccessful. The institutional intrapartum stillbirth rate was defined as the proportion of all facility births that resulted in intrapartum stillbirth. Training results were the proportion of total test items (knowledge questions or skill steps) performed correctly.

#### Statistical analysis

We ran a simulation to assess statistical power to detect a reduction in the primary outcome, 24-h newborn mortality, in the original 42 study sites. In each simulation run, the study design was replicated, with four study entry time points (i.e., strata) for 42 facilities. A random effect Poisson model was fit to each study replicate to estimate the intervention effect on the number of newborn deaths, adjusting for the stratum, with the number of deliveries per facility as the offset term. Across the multiple iterations of the study, we calculated the empirical statistical power as the proportion of the simulation runs in which the *p*-value was less than 0·05 level of significance, indicating that the null hypothesis of no post-intervention difference in the number of deaths is rejected. Results indicated that we had 99% power to detect a 25% decrease in the number of newborn deaths after the intervention.

We assessed institutional mortality rates and SBA competency at all facilities post-implementation compared to pre-implementation and assessed differences between institutional mortality rates at a subset of intervention and non-intervention (i.e., control) facilities during three 6-month time intervals. The entire study period (March 2014 to February 2017) was subdivided into four intervention waves, based on the start date of the intervention, and six calendar periods. For each facility the following variables were defined: intervention stage (coded as 0 for baseline, 1 for months 1–6 after Low-Dose 1, and 2 for months 7–12 after Low-Dose 1), wave (from 1 to 4, depending on the start of the intervention), and calendar period (from 1 to 6). Exploratory analyses included comparison of the observed intrapartum stillbirth or newborn death rates by intervention stage, wave, calendar period, and region. Outcome rate trajectories were plotted over the calendar period by wave to assess for within-group trends. Baseline mortality rates were calculated as a facility-weighted *(births or live births)* average over the 6-month baseline period. Comparability of mortality rates at baseline was assessed using analysis of variance (ANOVA).

To estimate the effect of the intervention, we fit a series of generalized population-average linear models with negative binomial distribution and a log-link with exchangeable working correlation structure. The models were estimated using generalized estimating equations with robust variance estimates and a negative binomial distribution.

The base model included two indicator variables for intervention (months 1–6 and months 7–12) and adjustment for the region and level of the health facility (regional hospital or district hospital/polyclinic). Exponentiated beta coefficients for the two intervention variables were interpreted as the relative risk (RR) of the outcome post-intervention compared to the historical control (baseline) period.

To assess whether the effect of the intervention varied significantly by wave or region, the base model was modified to include the appropriate interaction terms. Secondary analyses were conducted using time as a continuous variable in a similar model to assess the monthly change in mortality rates prior to and after intervention. In addition, we used interaction terms in a modified model to assess the effect of the intervention comparing intervention and control facilities in the same calendar period, adjusting for effect of facility level on the outcome measures.

OSCE and knowledge test scores were calculated based on the number of steps correct in the OSCE or number of items correct on the knowledge test out of the total possible correct on each test. Scores were analyzed using ordinary least-squares linear regression that tested for a difference in individuals’ pre- and post-test assessment scores, and pre-test and 1-year assessment scores, adjusting for clustering of SBAs within the facility.

Data were analyzed using Stata Version 14 (StataCorp LLC, College Station, TX, USA).

## Results

**Fig. 3:**
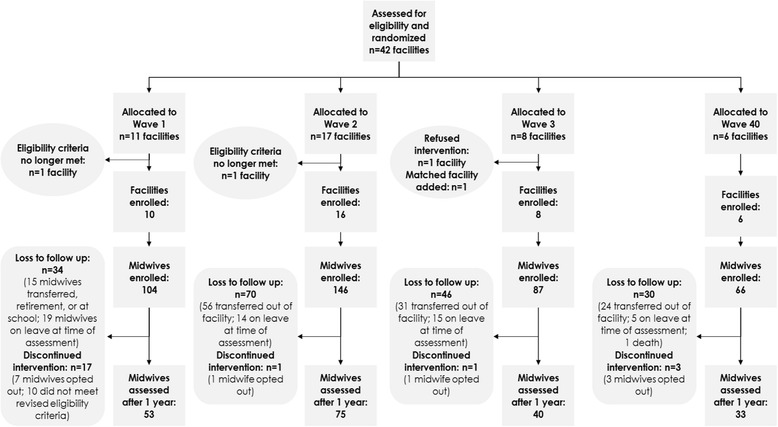
Trial profile. Describes the allocation of trial facilities into the four waves, the number of midwives enrolled in the trial and the number assessed after 1 year of implementation

In the 40 facilities, 403 SBAs consented and were enrolled in the study. Two hundred and one SBAs (50%) were available for OSCE and knowledge assessment at 1 year after the intervention due to SBA rotation to other facilities, further education, and annual leave (Fig. 3).

The pre-intervention period included 38,192 births (Table 3). Months 1–6 and months 7–12 included 36,160 and 31,498 births, respectively. The total numbers of 24-h newborn deaths and intrapartum stillbirths in the 40 sites, including baseline and the intervention periods, were 528 and 799, respectively.

**Table 3:** Outcomes of interest during pre- and post-intervention periods

	Pre-intervention	Post-intervention	
	Baseline	Months 1–6	Months 7–12
Number of births	38,192	36,160	31,498
Number of live births	37,204	35,352	30,940
Number of intrapartum stillbirths	392	242	165
Number of newborn deaths within 24 h of birth	284	140	104
Intrapartum stillbirth rate (per 1000 births)	10·3	6·7	5·2
24-h newborn mortality rate (per 1000 live births)	7·6	4·0	3·4

Delivery caseloads, intrapartum stillbirth rates, and 24-h newborn mortality rates prior to intervention were comparable across waves (ANOVA *p* = 0·818 and *p* = 0·527, respectively) (Table 4).

**Table 4:** Baseline facility characteristics

	Wave				ANOVA *p*-value
	1	2	3	4	
	Mean (+/− SD)	Mean (+/− SD)	Mean (+/− SD)	Mean (+/− SD)	
Number of births per month	194 (99)	117 (70)	136 (58)	110 (42)	0·0628
Births per month per participant trained	25 (18)	13 (8)	13 (7)	10 (4)	0·0336
Intrapartum stillbirth rate	9·0 (6·0)	10·7 (6·3)	12·0 (9·7)	12·0 (9·1)	0·818
24-h newborn mortality rate	11·7 (10·4)	8·2 (8·3)	5·9 (6·4)	7·0 (7·4)	0·527

Implementation of the LDHF package resulted in a 55% reduction in risk of newborn mortality within 24 h of birth in the 6 months after LD1 (unadjusted RR 0·45, 95% CI 0·35–0·58; *p* < 0·001) and a 65% reduction in risk in the next 6-month follow-up period (unadjusted RR 0·35, 95% CI 0·24–0·50; *p* < 0·001). After adjusting for region and facility level, the reduction in risk increased to 59% and 70%, respectively (adjusted RRs 0·41, 95% CI 0·32–0·51 and 0·30, 95% CI 0.21–0.43, in months 1–6 and months 7–12, respectively). Regional-level facilities had a significantly greater risk of 24-h newborn mortality compared to district-level facilities and polyclinics (RR 5·11, 95% CI 3·55–7·36; *p* < 0·001).

The intervention resulted in a 35% reduction (RR 0·65, 95% CI 0·54–0·78; *p* < 0001) in the risk of intrapartum stillbirth (unadjusted analysis) comparing months 1–6 to the pre-intervention period, and a 51% reduction in risk (RR 0·49, 95% CI 0·36–0·65; *p* < 0·001) in months 7–12 (Table 5). Results in a model adjusting for level of facility (regional vs. district level or polyclinic) and region did not substantially differ compared to the unadjusted model. Regional-level facilities had a significantly greater risk of intrapartum stillbirth compared to district-level facilities and polyclinics (RR 1·33, 95% CI 1·03–1·70; *p* = 0·027).

**Table 5:** Mortality outcomes in 40 sites

	Unadjusted risk ratio	95% CI	*p*-value	Adjusted risk ratio^a^	95% CI	*p*-value
Pre-intervention (6 months prior to intervention)	REF	REF	REF	REF	REF	REF
Newborn death within 24 h						
Months 1–6	0·45	0.35–0.58	< 0·001	0·41	0·32–0·51	< 0·001
Months 7–12	0·35	0.24–0.50	< 0·001	0·30	0·21–0·43	< 0·001
Intrapartum stillbirth						
Months 1–6	0·65	0·54–0·78	< 0·001	0·64	0·53–0·77	< 0·001
Months 7–12	0·49	0·36–0·65	< 0·001	0·48	0·36–0·63	< 0·001

*OSCE* objective structured clinical examination

^a^Adjusted for region and facility level (polyclinic or district hospital vs. regional hospital)

Pre-test scores on the low-dose session 1 (LD1) knowledge test and OSCE were 76% and 44%, respectively (Table 6). Low-dose session 2 (LD2) pre-test scores were slightly higher at 91% and 52%, respectively. Post-test scores increased by 11% (95% confidence interval [CI] 9–12; *p* < 0·001) and 4% (95% CI 2–6; *p* = 0·001) on LD1 and LD2 knowledge tests, respectively, and 44% (95% CI 40–48; *p* < 0·001) and 40% (95% CI 37–43; *p* < 0·001) on the LD1 and LD2 OSCEs, respectively. After 1 year, participants retained most knowledge and skills gains. Participants scored 31% and 28% higher on OSCE content from LD1 and LD2 at 1 year, respectively, compared to pre-test scores (LD1 95% CI 27–36; *p* < 0·001; LD2 95% CI 25–32; *p* < 0·001), and 8% and 2% higher, respectively, on knowledge content from LD1 and LD2 at 1 year, compared to pre-test scores (LD1 95% CI 6–10; *p* < 0·001; LD2 95% CI 0–4; *p* = 0·025).

**Table 6:** Skilled birth attendant retention of key knowledge and skills

	Pre-test (%)	Post-test (%)	Difference (%) (post−/pre-)	*p*-value	1-year assessment (%)	Difference (%) (1-yr/pre-)	*p*-value
	Mean (+/− SD)	Mean (+/− SD)	Mean (95% CI)		Mean (+/− SD)	Mean (95% CI)	
Low-dose session 1							
Knowledge assessment	76 (9)	87 (7)	11 (9–12)	< 0·001	85 (8)	8 (6–10)	< 0·001
OSCE	44 (13)	88 (9)	44 (40–48)	< 0·001	76 (14)	31 (27–36)	< 0·001
Low-dose session 2							
Knowledge assessment	91 (10)	95 (6)	4 (2–6)	0·001	94 (10)	2 (0–4)	0·025
OSCE	52 (14)	92 (7)	40 (37–43)	< 0·001	81 (12)	28 (25–32)	< 0·001

There was no evidence of a temporal trend in intrapartum stillbirth or 24-h newborn mortality rates in study sites before the intervention. Therefore, the model did not adjust for calendar time. Once the intervention began, newborn mortality declined significantly from 1 month to the next over the one-year intervention period. Intrapartum stillbirth rates did not change significantly on a monthly basis; from this, we infer that the reduction in stillbirth rate was more drastic and immediate following the start of the intervention, and remained low for the remainder of the one-year follow-up period. Wave 3 facilities showed a greater reduction in risk of 24-h newborn mortality during months 7–12 post-intervention compared to facilities in the other three waves. Risk of intrapartum stillbirth decreased more dramatically in wave 2 facilities compared to other facilities (Additional files 1 and 2: Tables S1 and S2). We found no significant difference in the mortality rates as a result of a master mentor being present at a study site.

We tested to see if there is a difference in the mortality rates between cohorts of facilities in the intervention phase and the baseline phase during the same calendar period. In all three time periods analyzed, newborn mortality rates were significantly lower in facilities in the intervention phase compared to those in the baseline phase; intrapartum stillbirth rates were also lower but only statistically significant in two of the three analyzed calendar periods. This analysis was conducted for a subset of the total study time and outcome observations, as the study was not designed or resourced to conduct a full randomization with comparison sites throughout the study duration.

## Discussion

The primary aim of the study was to assess newborn outcomes. Results showed significant reduction in intrapartum stillbirth and newborn death within 24 h. This could indicate that the quality of care during labor was sufficient to maintain maternal and fetal well-being. In addition, since resuscitation was attempted for all non-macerated newborns not breathing at birth, many babies who would otherwise have been declared intrapartum stillbirths but actually had intrapartum-related asphyxia, were successfully resuscitated [11] corroborating the assertion by Wall et al. that training SBAs in newborn resuscitation could prevent up to 30% of mortality in full-term babies due to intrapartum-related events [12]. Reduction in newborn deaths within 24 h may be attributed to the use of newborn care interventions for every baby (immediate drying and warming, skin-to-skin-contact, and immediate and exclusive breastfeeding) as well as rapid recognition and appropriate management of intrapartum-related asphyxia.

A second aim of the study was to assess the effect of the LDHF package on retention of key knowledge and skills after 1 year. We found that SBAs retained most knowledge and skills gained during low-dose sessions, which contributed to their ability to apply appropriate clinical interventions for improved newborn outcomes. The expectation after the low-dose sessions was that SBAs’ skills should not be lost after training, but maintained or even improved. The lowest 1-year OSCE score occurred in relation to LD1, which dealt with care during normal labor and birth and newborn resuscitation. This implies that although SBAs attended many births, their practices did not reflect the evidence-based approaches presented during the first low-dose session. Thus, they might have needed more practice in simulated and clinical settings in the updated skills and/or reinforcement by PPCs or master mentors. Similarly, the skills for newborn resuscitation were new for most SBAs, and since resuscitation is a comparatively rare event, there may have been a need for reinforcement over time to maintain them. This is consistent with evaluations of maintenance of newborn resuscitation skills during which SBAs had the opportunity to review and practice skills on a regular basis after training [13].

In addition, our results demonstrate the impact and importance of an integrated package of evidence-based, clinical interventions that considers the continuum of care for the mother and baby on the day of birth. The components of the LDHF approach were chosen to target the major global causes of intrapartum-related events that are associated with up to 42% of maternal mortality, 32% of stillbirths, and 23% of newborn mortality [14]. They included cross-cutting elements of care that can improve maternal and newborn outcomes, such as respectful maternity care, which includes encouragement of continuous support during labor, ambulation, and positions of choice for labor and birth. These have been shown to decrease the duration of labor and the use of cesarean or assisted delivery [15, 16]. Key evidence-based infection prevention practices were introduced during LD1 to decrease the risk of maternal and newborn sepsis. Hand hygiene, [17] instrument processing, [18] reduction of vaginal exams during labor, [19] avoiding routine rupture of membranes, [20] and prompt management of maternal fever and newborn sepsis were emphasized [21]. Severe pre-eclampsia and eclampsia are implicated globally in up to 4·7% of stillbirths, with the highest burden in south Asia and sub-Saharan Africa. Improved management of these disorders during labor could avert these deaths [22, 23]. Respectful maternity care and infection prevention were part of all OSCEs and skills checklists used by providers during training and high-frequency practice sessions.

The study emphasized correct use of the partograph to monitor labor and support clinical decision-making, which has been shown to improve identification of prolonged labor, a major contributor to perinatal mortality [24]. Best practices for immediate newborn care included immediate drying and skin-to-skin contact, [25, 26] delayed cord clamping, [27] and breastfeeding in the hour following birth [25].

Leading causes of maternal mortality in sub-Saharan Africa include hemorrhage (25%), hypertension (16%), and sepsis (10%) [5]. Data for Ghana indicate that hemorrhage causes 24·3% of maternal mortality, hypertension accounts for 8·5%, and sepsis is the cause of 6·7% of deaths [28]. Thus, the LDHF approach targeted management of severe pre-eclampsia/eclampsia, using magnesium sulfate and antihypertensive medications, [29] and prevention and treatment of postpartum hemorrhage [30].

One of the factors contributing to effective implementation of the LDHF approach was close collaboration with all levels of the Ghana Health Service. National-level support was obtained at the outset, and regional health directors agreed to implementation in their regions. They also selected the MMs who carried out study activities as part of their normal duties; no MMs or SBAs were hired for the study. The investment in the development of a cadre of ten MMs in each region enabled close local follow-up of PPCs and SBAs in study facilities, both face-to-face and via mMentoring.

This study provides evidence that can guide strategies for health worker capacity-building at scale. Our results show a plausible causative association between use of an LDHF approach for facility-based in-service training and significant reductions in intrapartum stillbirths and newborn deaths in health facilities in low-resource settings. Health facilities did not undergo structural changes or receive additional equipment beyond delivery sets and resuscitation equipment. The LDHF approach built SBAs’ competency in essential maternal and newborn health care practices. In each facility, all SBAs providing intrapartum and immediate postpartum care participated in this approach, resulting in facility-wide improvement in service quality, while minimizing the amount of time the SBAs spent outside of their facilities. It also enabled nearly all SBAs in each facility to receive the same training and follow-up support, which may have enhanced consistency of knowledge and skill transfer to the clinical setting. The critical mass of trained SBAs at each facility reinforces learning and builds a system of checks and balances throughout the health facility, likely rendering the change more sustainable. Coupled with ongoing practice and mentorship, improved practices are sustained and health outcomes continue to improve over time, even with staff attrition and turnover, as new SBAs are taught the skills by existing staff and can practice them on the simulators. Moreover, a pool of highly competent MMs enables continued application of the package after the study ends.

There are methodological limitations to consider in assessing the study implications. Because the study was embedded in program implementation, facility staff were aware of the outcome of interest, which could have affected their actions and performance. The presence of MMs at some facilities before intervention start-up could also have affected baseline outcome measurement rates. However, our analysis shows that mortality rates in all waves were comparable at baseline, and therefore we do not believe this has an effect on the results achieved. In addition, our study was not designed to be able to conduct a full time-series or randomized control trial analysis, due to resource limitations on baseline and control site data collection. Multiple analyses conducted using available data demonstrate qualitatively the same result. Other sources of potential bias include lack of concealment in group (i.e., wave) assignment and information bias such that data quality may have improved after the intervention started. In all likelihood, improved data quality following the intervention would result in more deaths being reported, rather than fewer, resulting in a smaller observed effect.

Although the regions for study implementation were chosen specifically to avoid overlap with other interventions conducted by the GHS and nongovernmental organizations, some SBAs from study facilities were invited to attend training on content related to BEmONC and/or newborn resuscitation, but none of the training was similar to the LDHF approach. However, the influence of these activities on our outcomes cannot be ruled out.

Finally, while the technical approach demonstrates results, other factors such as cost and human resource availability (for trainers and mentors) must be considered. A nested costing study showed that the LDHF package was less expensive per participant compared to the traditional approach [31].

## Conclusions

Implementation of an LDHF approach to train SBAs in key evidence-based intrapartum and immediate newborn care practices is associated with a significant reduction in the risks of intrapartum stillbirth and newborn mortality within 24 h of birth. The components of the approach were chosen specifically to address the most common causes of intrapartum stillbirth and early newborn death in Ghana and were reinforced in multiple ways: classroom learning and practice with simulators during and between low-dose sessions; peer-led practice and feedback between and after low-dose sessions; follow-up via targeted SMS messaging and support by a master mentor; and robust data collection and reporting. In addition, all training was done onsite and involved nearly all maternity ward SBAs at each site. These elements contributed to transfer of knowledge and skills to the clinical setting and thus to improved outcomes. The adoption of the LDHF approach in Ghana and other low-resource countries has the potential to contribute to the acceleration of newborn survival and the achievement of Sustainable Development Goal 3 (reduction of neonatal mortality to at least as low as 12 per 1000 by 2030).

Policymakers and health partners should review current approaches to improving service delivery in maternal and newborn health, and consider investing in and adopting this approach as an innovative and effective method of training health care workers. While further research is necessary, we believe this approach should be considered for the improvement of clinical services in other technical areas that mandate acquisition and retention of skills to improve health outcomes.

## Additional files

Additional file 1: **Table S1.** Newborn mortality risk ratio by wave. Describes the effect of the intervention on newborn mortality by intervention wave. (DOCX 15 kb)

Additional file : **Table S2.** Intrapartum stillbirth risk ratio by wave. Describes the effect of the intervention on intrapartum stillbirth by intervention wave. (DOCX 15 kb)

## Abbreviations

ANOVA: Analysis of variance
BEmONC: Basic emergency obstetric and newborn care
CI: Confidence interval
GHS: Ghana Health Service
LD1: Low dose session 1
LD2: Low dose session 2
LDHF: Low dose, high frequency
MM: Master mentor
OSCE: Objective structured clinical examination
PPC: Peer practice coordinator
RR: Relative risk
SBA: Skilled birth attendant
SMS: Short message service
U5: Under 5

## Footnotes

1 UN Sustainable Development Goals (http://www.un.org/sustainabledevelopment/health/page/2/0; accessed 26 January 2017)

2 A skilled attendant is an accredited health professional such as a midwife, doctor, or nurse who has been educated and trained to proficiency in the skills needed to manage normal (uncomplicated) pregnancies, childbirth, and the immediate postnatal period, and in the identification, management, and referral of complications in women and newborns. (WHO, ICM and FIGO. *Making pregnancy safer: the critical role of the skilled attendant. A joint statement by WHO, ICM and FIGO*. Geneva, 2004.)

3 Project Fives Alive! is implemented by the Institute for Healthcare Improvement and the National Catholic Health Service in collaboration with the Ghana Health Service.
