# Supplementary material for: Accelerating newborn survival in Ghana through a low-dose, high-frequency health worker training approach: a cluster randomized trial
Source: BMC Pregnancy Childbirth. 2018 Mar 22;18:72. doi: 10.1186/s12884-018-1705-5 (PMC5863807; doi:10.1186/s12884-018-1705-5)
Supplement: Supplementary file 1 — Table S1. Newborn mortality risk ratio by wave. Describes the effect of the intervention on newborn mortality by intervention wave. (DOCX 15 kb) [file 12884_2018_1705_MOESM1_ESM.docx]

**Table S1: Newborn mortality risk ratio by wave**

|  | **Adjusted risk ratio*** | **95% CI** | **p-value** |
| --- | --- | --- | --- |
| **Wave 1 (Sept. 2014–Aug. 2015)** | | | |
| Pre-intervention  (6 months prior to intervention) | REF | REF | REF |
| Months 1–6 | 0·45 | 0·28–0·72 | 0·001 |
| Months 7–12 | 0·36 | 0·19–0·68 | 0·002 |
| **Wave 2 (Mar. 2015–Feb. 2016)** | | | |
| Pre-intervention  (6 months prior to intervention) | REF | REF | REF |
| Months 1–6 | 0·42 | 0·29–0·60 | <0·001 |
| Months 7–12 | 0·35 | 0·19–0·64 | 0·001 |
| **Wave 3 (Sept. 2015–Aug. 2016)** | | | |
| Pre-intervention  (6 months prior to intervention) | REF | REF | REF |
| Months 1–6 | 0·29 | 0·17–0·48 | <0·001 |
| Months 7–12 | 0·11 | 0·06–0·21 | <0·001 |
| **Wave 4 (Mar. 2016–Feb. 2017)** | | | |
| Pre-intervention  (6 months prior to intervention) | REF | REF | REF |
| Months 1–6 | 0·42 | 0·30–0·60 | <0·001 |
| Months 7–12 | 0·24 | 0·11–0·50 | <0·001 |

*Adjusted for region and facility level (polyclinic or district hospital vs. regional hospital)
