# Supplementary material for: Accelerating newborn survival in Ghana through a low-dose, high-frequency health worker training approach: a cluster randomized trial
Source: BMC Pregnancy Childbirth. 2018 Mar 22;18:72. doi: 10.1186/s12884-018-1705-5 (PMC5863807; doi:10.1186/s12884-018-1705-5)
Supplement: Supplementary file 2 — Table S2. Intrapartum stillbirth risk ratio by wave. Describes the effect of the intervention on intrapartum stillbirth by intervention wave. (DOCX 15 kb) [file 12884_2018_1705_MOESM2_ESM.docx]

**Table S2: Intrapartum stillbirth risk ratio by wave**

|  | **Adjusted risk ratio*** | **95% CI** | **p-value** |
| --- | --- | --- | --- |
| **Wave1** | | | |
| Pre-intervention  (6 months prior to intervention) | REF | REF | REF |
| Months 1–6 | 0·80 | 0·59-1·09 | 0·161 |
| Months 7–12 | 0·76 | 0·57-1·00 | 0·050 |
| **Wave 2** | | | |
| Pre-intervention  (6 months prior to intervention) | REF | REF | REF |
| Months 1–6 | 0·45 | 0·32-0·63 | <0·001 |
| Months 7–12 | 0·30 | 0·19-0·48 | <0·001 |
| **Wave 3** | | | |
| Pre-intervention  (6 months prior to intervention) | REF | REF | REF |
| Months 1–6 | 0·85 | 0·66-1·10 | 0·209 |
| Months 7–12 | 0·61 | 0·39-0·95 | 0·030 |
| **Wave 4** | | | |
| Pre-intervention  (6 months prior to intervention) | REF | REF | REF |
| Months 1–6 | 0·55 | 0·40-0·64 | <0·001 |
| Months 7–12 | 0·16 | 0·08-0·33 | <0·001 |

*Adjusted for region and facility level (polyclinic or district hospital vs. regional hospital)
